# Isolation of Dihydroflavonol 4-Reductase cDNA Clones from *Angelonia* x *angustifolia* and Heterologous Expression as GST Fusion Protein in *Escherichia coli*

**DOI:** 10.1371/journal.pone.0107755

**Published:** 2014-09-19

**Authors:** Christian Gosch, Karthik Mudigere Nagesh, Jana Thill, Silvija Miosic, Sylvia Plaschil, Malvina Milosevic, Klaus Olbricht, Shaghef Ejaz, Annette Rompel, Karl Stich, Heidi Halbwirth

**Affiliations:** 1 Vienna University of Technology, Institute of Chemical Engineering, Vienna, Austria; 2 Julius Kühn-Institut, Institute for Breeding Research on Horticultural Crops, Quedlinburg, Germany; 3 University of Vienna, Department of Biophysical Chemistry, Vienna, Austria; 4 Humboldt University Berlin, Institute of Agriculture and Horticulture, Berlin, Germany; 5 Bahauddin Zakariya University, Department of Horticulture, Multan, Pakistan; Indiana University, United States of America

## Abstract

Blue *Angelonia × angustifolia* flowers can show spontaneous mutations resulting in white/blue and white flower colourations. In such a white line, a loss of dihydroflavonol 4-reductase (DFR) activity was observed whereas chalcone synthase and flavanone 3-hydroxylase activity remained unchanged. Thus, cloning and characterization of a *DFR* of *Angelonia* flowers was carried out for the first time. Two full length *DFR* cDNA clones, *Ang.DFR1* and *Ang.DFR2*, were obtained from a diploid chimeral white/blue *Angelonia × angustifolia* which demonstrated a 99% identity in their translated amino acid sequence. In comparison to *Ang.DFR2*, *Ang.DFR1* was shown to contain an extra proline in a proline-rich region at the N-terminus along with two exchanges at the amino acids 12 and 26 in the translated amino acid sequence. The recombinant Ang.DFR2 obtained by heterologous expression in yeast was functionally active catalyzing the NADPH dependent reduction of dihydroquercetin (DHQ) and dihydromyricetin (DHM) to leucocyanidin and leucomyricetin, respectively. Dihydrokaempferol (DHK) in contrast was not accepted as a substrate despite the presence of asparagine in a position assumed to determine DHK acceptance. We show that substrate acceptance testing of DFRs provides biased results for DHM conversion if products are extracted with ethyl acetate. Recombinant Ang.DFR1 was inactive and functional activity could only be restored via exchanges of the amino acids in position 12 and 26 as well as the deletion of the extra proline. *E. coli* transformation of the pGEX-6P-1 vector harbouring the *Ang.DFR2* and heterologous expression in *E. coli* resulted in functionally active enzymes before and after GST tag removal. Both the GST fusion protein and purified DFR minus the GST tag could be stored at −80°C for several months without loss of enzyme activity and demonstrated identical substrate specificity as the recombinant enzyme obtained from heterologous expression in yeast.

## Introduction

**Figure 1:**
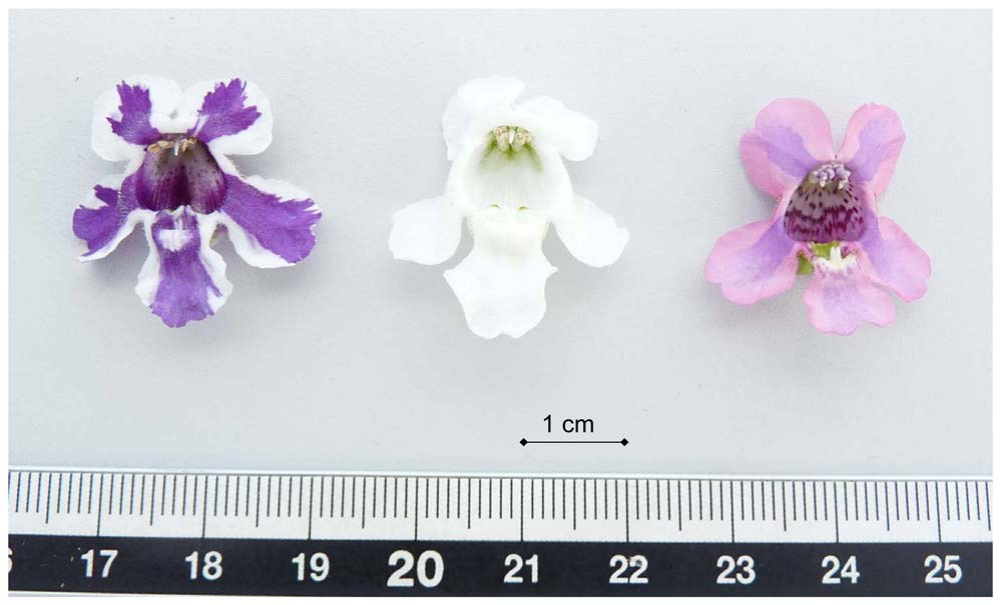
*Angelonia* × *angustifolia* flowers.

The genus *Angelonia* is a perennial plant which originates from South America. It belongs to the family Plantaginaceae (formerly Scrophulariaceae) [1], which comprises about 30 species, displaying a great diversity in form and colour ranging from blue to violet, white and pink (Figure 1) [2], [3]. The background of existing cultivar species is neither correctly reported nor investigated so far. *Angelonia salicariifolia* Humb. et Bonpl. (syn. *Angelonia salicariaefolia* Humb. et Bonpl., syn. *Angelonia gardnerii* Hook.), *Angelonia angustifolia* Benth., *Angelonia grandiflora* C. Morr., *Angelonia integerrima* Spreng. are currently considered as ancestral species [4]–[6]. We therefore use the term *Angelonia* × *angustifolia*. *A.* × *angustifolia* is sold as a flowering pot and bedding plant. It also has a high potential as a cut flower [7]. Spontaneous mutations occur which result in bicoloured flowers segregating in white and blue genotypes [2]. The bicolourness in *Angelonia* flowers is caused histogenetically (chimeral flower patterns) [2]. This makes it an interesting object for studies on the underlying molecular mechanisms.

**Figure 2:**
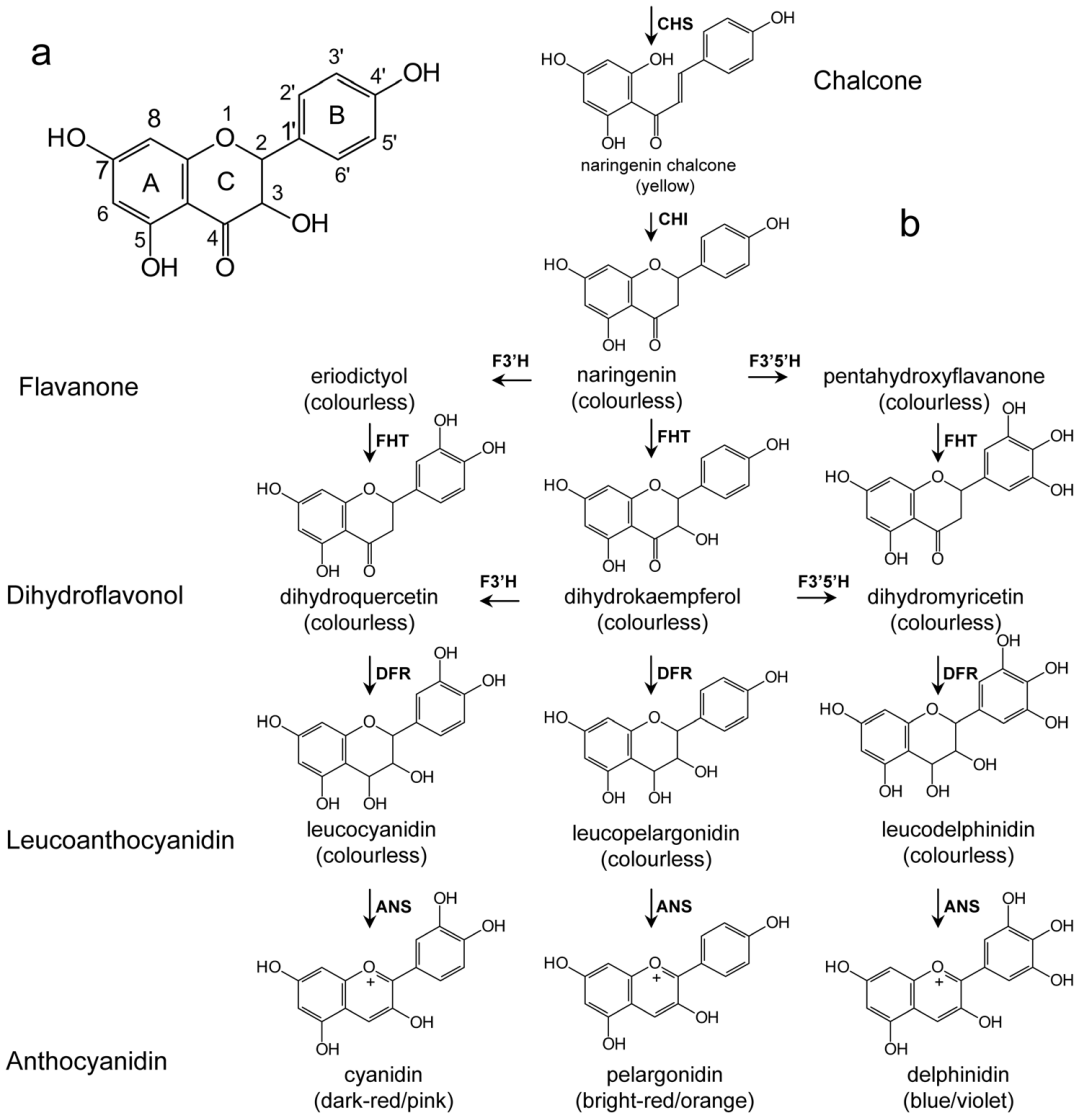
a: Chemical structure of dihydrokaempferol showing ring denotation and atom numbering. b: Simplified overview of the anthocyanin pathway. abbrev: ANS: anthocyanidin synthase, CHI: chalcone isomerase, CHS: chalcone synthase, DFR: dihydroflavonol synthase, FHT: flavanone 3-hydroxylase, F3′H: flavonoid 3′-hydroxylase, F3′5′H: flavonoid 3′,5′-hydroxylase.

In species which usually accumulate anthocyanins as plant pigments, white flowering genotypes result from a deficiency in at least one step of the anthocyanin pathway [8]. Such blockage of the anthocyanin pathway leading to white flower colour may occur at several levels. Deficiency in the activities of chalcone synthase (CHS), flavanone 3-hydroxylase (FHT), dihydroflavonol 4-reductase (DFR) and anthocyanidin synthase (ANS) results in the accumulation of their colourless upstream intermediates (Figure 2). Frequently DFR and/or FHT are affected [9]–[16]. In contrast, absence of flavonoid 3′-hydroxylase (F3′H) and flavonoid 3′,5′-hydroxylase (F3′5H) activities only results in a shift of flower colour as this changes the anthocyanin spectrum in the flower (Figure 2). A lack of enzyme activity can be based on mutations in the structural gene leading to a loss of functional activity or regulatory processes at the transcriptional or post-transcriptional level [17], [18]. Post-transcriptional regulation processes are less understood, but micro RNAs seem to be largely involved [19]–[24]. At the transcriptional level, a variety of factors may result in lacking gene expression including mutations or transposable element insertions in structural genes and promoter regions, differentially expressed transcriptional factors and gene methylation.

The establishment of flower colour can also be influenced by the substrate specificity of the enzymes, particularly by DFR [25], [26]. DFR is an oxidoreductase (EC 1.1.1.219) catalyzing the NADPH dependent stereospecific reduction of (+)-(*2R,3R*)-dihydroflavonols to the respective (*2R,3S,4S*)-flavan-2,3-*trans*-3,4-*cis*-diols (leucoanthocyanidins), as well as the reverse reaction in the presence of NADP^+^ [27], [28]. DFR is the first of the so-called ‘late’ enzymes of the flavonoid pathway which shows a major impact on the formation of anthocyanins, flavan 3-ols and flavonols. As a rule, DFRs accept dihydroflavonols independently from their hydroxylation pattern in the *B*-ring. Dihydrokaempferol (DHK, one hydroxyl group), dihydroquercetin (DHQ, two hydroxyl groups) and dihydromyricetin (DHM, 3 hydroxyl groups) are thus converted into leucopelargonidin, leucocyanidin and leucomyricetin, respectively. This provides bright-red pelargonidin, dark-red/pink cyanidin and blue/violet delphinidin possessing one, two and three hydroxyl groups, respectively in the *B*-ring (Figure 2). The DFR of some plants, however, show substrate specificity by accepting only dihydroflavonols showing at least two hydroxyl groups in the *B*-ring for example DFR from *Petunia* x *hybrida*, *Nicotiana tabacum* and *Cymbidium hybrida* [16], [29].

We analyzed cyanic and acyanic flowers of *Angelonia* for the absence of enzymes from the anthocyanin pathway and demonstrated that the acyanic line is DFR deficient. For future studies into the molecular basis behind flower colour variegation in *Angelonia* we isolated for the first time *DFR* cDNA clones of this plant species. This study provides important clues for the investigation of DFR characteristics in general.

## Results and Discussion

### Enzyme activity in *Angelonia × angustifolia* with divergent flower colour

Three key enzymes of the flavonoid pathway were tested in chimeral pink/blue, chimeral white/blue and homohistic white flowers of *A. × angustifolia*: CHS/CHI, FHT and DFR. ANS could not be included because demonstrations in crude plant preparations failed so far [10] (and unpublished results) and were, therefore, only studied with recombinant and/or purified enzymes [30]. Two different protocols [31] for the preparation of enzyme extracts from the flowers were tested. All three enzymes could be detected with preparations from flowers which were obtained according to a relatively simple extraction procedure with quartz sand and buffer, although with only moderate activity (Table 1). CHS and DFR activity were slightly higher when the preparations were obtained via a protocol particularly developed for polyphenol rich tissues [31], [32] (data not shown). Whereas CHS/CHI and FHT activity was comparable in all three flower colour types, no DFR activity could be measured in white flowers in contrast to the white/blue and pink/blue flowers (Table 1). Heat inactivated enzyme preparations did not show any enzyme activity.

**Table 1:** Specific activities [nmol/s*kg] of chalcone synthase/chalcone isomerase (CHS/CHI), flavanone 3-hydroxylase (FHT) and dihydroflavonol 4-reductase (DFR) in genotypes of *Angelonia* × *angustifolia* flowers showing divergent flower colour.

Colour of Angelonia × angustifolia flower	Specific enzyme activity [nmol/s*kg]		
	CHS/CHI	FHT	DFR
**pink/blue**	34±3	63±3	260±15
**white/blue**	56±4	44±2	125±10
**white**	64±5	48±4	0

### Cloning of DFR from Angelonia × angustifolia

Based on the *DFR* sequence information for the most closely related species available in the NCBI database, *Antirrhinum pallida*, *Mimulus aurantiacus*, *Forsythia × intermedia* and *Solenostemon scutellarioides*, degenerated primers (Table S1) were designed in conserved regions, which were used to isolate a fragment of a putative *DFR* cDNA clone from blue flowering petals of *A. × angustifolia*. Full sequence information was obtained by 5′ and 3′ RACE techniques (rapid amplification of cDNA ends) and specific primers were created (A-DFRfullF, A-DFRfullR, Table S1) which resulted in the isolation of two full size cDNA clones from the same plant material. *Ang.DFR1* (Accession No. KJ817183) consisted of 1317 bp with an open reading frame of 438 deduced amino acids and *Ang.DFR2* (Accession No. KF285561) of 1314 bp with an open reading frame of 437 deduced amino acids. Several attempts to isolate a *DFR* cDNA clone from the white line failed despite the common genetic background. This correlates with the absence of DFR activity in white lines and supports our hypothesis that DFR deficiency could be based on a lack of *DFR* expression.

**Figure 3:**
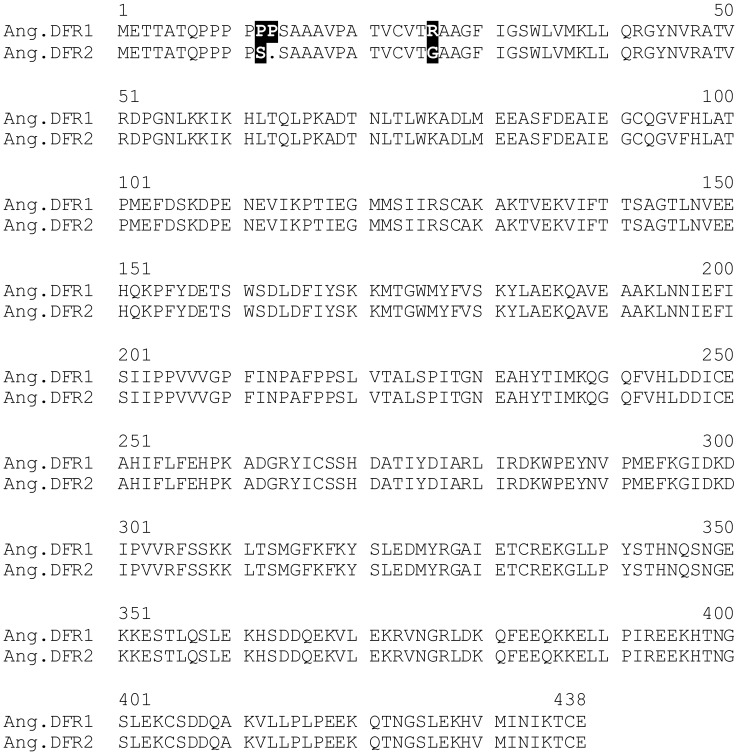
Alignment of the DFRs isolated from petals of *Angelonia* × *angustifolia*.

**Figure 4:**
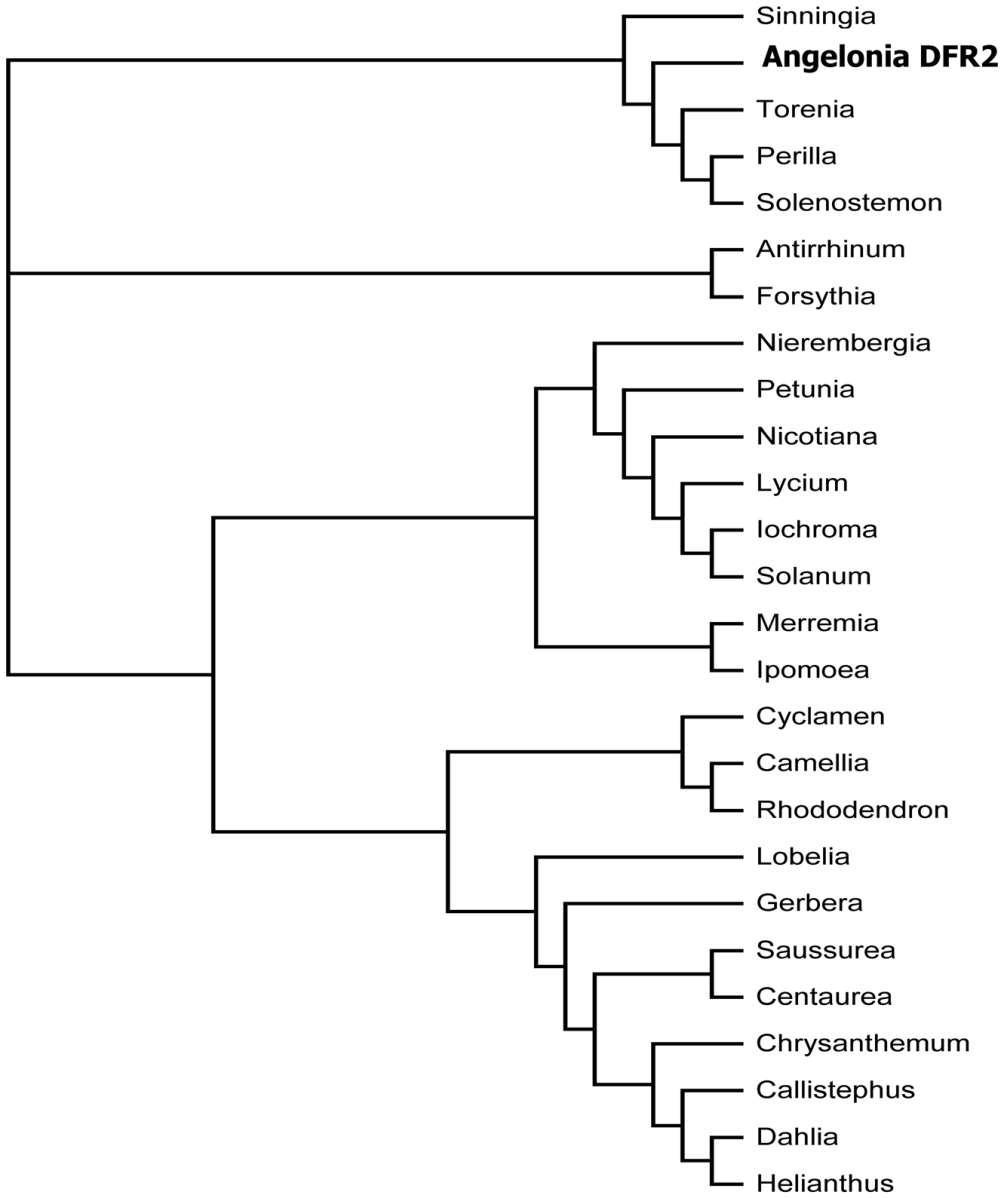
Phylogenetic tree of amino acid sequences of DFRs from different plant species. The following sequences were used (Accession numbers in parentheses): *Angelonia* × *angustifolia* Ang.DFR2 from the present study (KF285561), *Antirrhinum majus* (X15536), *Perilla frutescens* (AB002817), *Forsythia* × *intermedia* (Y09127), *Solenostemon scutellarioides* (EF522155), *Sinningia cardinalis* (AY332536), *Nierembergia sp.* (AB078510), *Torenia hybrida* (AB012924), *Camellia sinensis* (AB018686), *Petunia* × *hybrida* (EU189078), *Nicotiana alata* (FJ969389), *Iochroma cyaneum* (GU595064), *Solanum pinnatisectum* (AY954035), *Lycium ruthenicum* (JN849097), *Lobelia erinus* (AB221076), *Saussurea medusa* (EF600682), *Merremia dissecta* (EU189077), *Ipomoea purpurea* (AF028601), *Rhododendron simsii* (AJ413278), *Centaurea maculosa* (FJ376591), *C. chinensis* (Z67981), *Dahlia variabilis* (FJ216425), *Chrysanthemum* × *morifolium cultivar Shenyun* (JF346164), *Helianthus annuus* (EU095849), *Cyclamen graecum* (AB517921), *Gerbera hybrid* cv. ‘Terra Regina’ (Z17221).

The two cDNA clones shared a sequence identity of 99% at the nucleotide level. In comparison to *Ang.DFR1*, *Ang.DFR2* showed 4 exchanges and a deletion of 3 bp, which resulted in an exchange of two amino acids and a deletion of a proline in a proline-rich region at the N-terminus (Figure 3). Compared to many other *DFRs*, the cDNA clones from *Angelonia* were approximately 50 to 100 amino acids longer, but the length was comparable to those of the DFRs from the closely related species *Antirrhinum majus* (Accession X15536) and *Perilla frutescens* (Accession AB002817). In the phylogenetic analysis, the *Angelonia DFR*s clustered together with *DFRs* from *Torenia*, *Perilla*, *Solenostemon* and *Sinningia*, which all belong to the order Lamiales (Figure 4).

### Heterologous expression in yeast and characterization of the recombinant enzymes

The cDNA clones were transferred into the pYES2.1 vector and heterologously expressed in yeast (*Saccharomyces cerevisiae*). The recombinant Ang.DFR2 was functionally active catalyzing the NADPH dependent conversion of dihydroflavonols into leucoanthocyanidins (Table 2, grey-shaded). The enzyme demonstrated substrate specificity as described for a number of plants [25], [26] and accepted DHQ and DHM as substrates but did not efficiently convert DHK. Johnson et al. [25] demonstrated that substrate specificity of DFR is determined in the amino acid region 132–157 in the *Gerbera hybrida* DFR, which corresponds to amino acids 141–168 in Ang.DFR2. The presence of an aspartic acid in position 134 (position 143 of Ang.DFR2) of *Petunia hybrida* contrasts with the highly conserved asparagine found in many plant species and is suggested to be essential for the substrate specificity [25]. However, Ang.DFR2 showed the same substrate specificity as the *P. hybrida* DFR despite the presence of an asparagine in this position. Ang.DFR1 in contrast did not show functional activity with any dihydroflavonol tested (Table 2, grey-shaded). The recombinant Ang.DFR2 was characterized in detail and the result is summarized as a standard enzyme assay in the Material and Method section and in Table 3.

**Table 2:** Functional activity, substrate specificity and influence of mutations in the N-terminus on the functional activity of DFR from *Angelonia* × *angustifolia*, varying positions between the two DFR types are marked in bold.

		% Formation of		
	Mutation	LPg	LCy	LDp
Ang.DFR1	PPPPP **P** SAAAVPATVCVT **R** A	0	0	0
G21	PPPPP **P** SAAAVPATVCVT **G** A	0	0	0
G23	PPPP **S** SAAAVPATVCVT **R** A	0	0	0
G24	PPPP **S** SAAAVPATVCVT **G** A	0	53±2	47±3
Ang.DFR2	PPPP **S** SAAAVPATVCVT **G** A	0	50±4	46±3
G3	PPPP **S** SAAAVPATVCVT **R** A	0	0	0
G4	PPPPP **P** SAAAVPATVCVT **G** A	0	0	0
G128	PPPPP **P** SAAAVPATVCVT **R** A	0	0	0

**Table 3:** Characterization of recombinant DFR from *Angelonia* × *angustifolia* obtained from heterologous expression in yeast (left) and *E. coli* (right).

	Recombinant Ang.DFR2 from expression in yeast	Recombinant Ang.DFR2 from expression in *E. coli*
pH optimum	6.50	6.0
Temperature optimum [°C]	25	25
Temperature stability [°C]	25	25
Time linearity [min]	20	20
Protein linearity [µg in assay]	22	0.5
specific activity [µkat/kg protein]	0.41^1^	818^1^
apparent *K_m_* [µM]	11^1^/6.5^2^	not determined
*K_cat_/K_m_* [l/s*kg]	1.2^1^/0.9^2^	not determined

1 DHQ as a substrate,

2 DHM as a substrate.

### Testing DFR activities

Substrate specificity of DFR has been intensively studied in various plants [25], [26], [28], [33]–[41]. Substrate acceptance of DFR is either studied *in vitro* using recombinant enzymes, using enzyme preparations from plant sources, or *in planta* by monitoring changes in the flower colour of genetically modified plants. When substrate acceptance is compared *in vitro*, several sources of error exist. Firstly, it is essential to consider the stereospecificity of DFR, which accepts only (+)-(*2R,3R*)-dihydroflavonols as substrates [42]. Commercially available dihydroflavonols are frequently racemic mixtures and may occur in various qualities. The presence of impurities or the wrong stereoform may significantly hamper the reaction. Incubation of Ang.DFR2 with equimolar amounts of (+)-DHQ and a racemic mixture ((±)-taxifolin hydrate), respectively, reduced conversion rates of (±)-taxifolin by approximately 72% compared to (+)-DHQ. This is significantly higher than the 50% one would have expected from the fact that only one of the two stereoforms is converted. For comparison of different dihydroflavonols, it is therefore essential to use all of the same quality, stereoform and in equimolar amounts (same nmol, not µg). Another problem stems from the instability of leucoanthocyanidins, which are rapidly oxidised and polymerized and may react with proteins [43]–[49]. With an increasing number of hydroxyl groups, compounds show a higher sensitivity towards oxidation and polymerization. Losses may thus vary for the different substrates and this could bias conclusions on substrate preference. Long incubation times (hours) should therefore be avoided.

As a rule, substrates and products are extracted with ethyl acetate and conversion rates are determined after separation on TLC plates [37],[38],[50] or HPLC [33]. However solubility of leucoanthocyanidins in organic solvents varies [45] with their polarity, which increases with the number of hydroxyl groups. Compared to leucoanthocyanidins, dihydroflavonols are more easily extracted by ethyl acetate (Table S2). For DHK and DHQ this causes an acceptable error in conversion rates of approximately 5%. Solubility of leucodelphinidin decreases dramatically however when compared to DHM and the other leucoanthocyanidins (Table S2). As a result biased results are obtained for the conversion of DHM (Table 4). When leucodelphinidin was extracted with ethyl acetate, recovery rates were low in both organic and aqueous solvents (Table S2). We assume that this is a result of unspecific interactions with proteins in the interlayer after oxidation of the phenolic groups. Thus we recommend the separation of whole assays on paper stripes as described for glycosyltransferases [51]. This enables exhaustive recovery and reliable conversion rates for DHM.

**Table 4:** Dependence of product formation through sample handling after stopping the enzyme reactions.

	% Formation of		
Sample handling	LPg	LCy	LDp
no extraction, sample transferred on paper stripes	42±2	77±5	21±1
separation of ethyl acetate extracts on TLC plates	40±4	77±3	7±3
remaining aqueous phase after ethyl acetate extraction transferred on paper stripes	n.d.	77±4	53±3*
separation of 1-butanol extracts on TLC plates	43±2	75±2	9±2

The substrates were converted with recombinant dahlia DFR (Accession FJ216425). Substrates and products were separated on cellulose in chloroform/acetic acid/water (10∶9∶1, v:v:v). n.d: not detected.

*total amount of radioactivity was very low.

Some authors demonstrate functional activity of DFRs by conversion of the formed leucoanthocyanidins into anthocyanidins via acidic treatment and subsequent anthocyanin quantification with HPLC or via photometer [40], [41], [52]. However, the conversion is not quantitative and strongly depends on various factors [45]. Measurements of the converted anthocyanidin are therefore an acceptable tool for demonstrating functional activity of DFRs. It remains questionable however whether this provides reliable results for the comparison of substrates with divergent hydroxylation patterns.

### Heterologous expression as glutathione S-transferase (GST) fusion protein in *E. coli*

Heterologous expression of *DFR* is frequently performed in *S. cerevisiae* [50], however, this is time-consuming in comparison with expression in *E. coli*. Only a few recombinant *DFR*s were successfully expressed in *E. coli* thus far [28], [33], [40], [41], [53] with instability after His-tag removal reported. We therefore tested whether heterologous expression as a GST fusion protein in *E. coli* could be a viable option to rapidly produce highly purified, active recombinant DFR. The *DFR*s were subcloned from pYES2.1 into the pGEX-6P-1 vector and heterologously expressed in *E. coli*. Purification of the recombinant proteins was performed by using glutathione Sepharose^™^4B in either a batch procedure or by using GST Spin Traps. When the GST Spin Traps were used, a rapid loss of enzyme activity within two days was observed unless the enzyme was stabilized with BSA (2 mg/ml) and kept in Protein LoBind tubes. Both the resulting GST fusion protein and the purified enzyme after GST removal showed functional activity and no difference was observed in either the substrate acceptance of the fusion protein, the purified protein after GST removal or the recombinant enzyme produced in yeast. The purified recombinant DFR was stable and could be kept at −80°C for several months without loss of activity.

### Restoring the functional activity of Ang.DFR1 by site-directed mutagenesis

Although the two cDNA clones differed only in three amino acids, no functional activity could be observed with recombinant Ang.DFR1. To identify the decisive residues, we mutated the two *DFR* cDNA clones by site-directed mutagenesis and investigated the resulting recombinant proteins for functional activity. Neither the deletion of the extra proline, along with an exchange of serine to proline in the Ang.DFR1 sequence nor an exchange of the arginine 26 with glycine alone was able to restore the functional activity of Ang.DFR1. Only simultaneous mutation of all three positions resulted in a highly active recombinant Ang.DFR1. In the same way, exchange of glycine to arginine in Ang.DFR2 or insertion and exchange of an additional proline with serine at position 12 led to a complete loss of Ang.DFR2 activity (Table 2). The proline-rich area in the *Angelonia DFR* is closely located to the N-terminus and contains a unique structural feature (Figure S1), which is also not found in the *Vitis* DFR (Swiss-Prot Accession Number P93799_VITVI) and therefore not covered by the crystal structure [28]. It is thus difficult to explain why differences in the three amino acids are essential for functional activity. The region however, is closely located to a glycine-rich motif constituting the NADPH binding site [28]. As proline often acts as a structural disruptor [54] we theorize that the presence of two additional prolines and the large arginine residue in position 26 of Ang.DFR1, which contrasts with the small glycine in Ang.DFR2, has an negative impact on protein folding or disturbs proper NADPH binding.

## Conclusions

This study focused on the isolation and characterization of *DFR* from *Angelonia* x *angustifolia* which seems to play an essential role in the formation of chimeral patterns resulting in the formation of white/blue flowers. The DFR was used as model cDNA clone to demonstrate the suitability for heterologous expression as a GST fusion protein in *E. coli*. In addition, we demonstrated possible sources for biased results in substrate acceptance studies with DFRs.

## Materials and Methods

### Chemicals

(2-^14^C)-Malonyl-coenzyme A (55 mCi/mmol) was purchased from New England Nuclear Corp. GmbH (Vienna, Austria). (^14^C)-Labeled dihydroflavonols, (+)-DHK, (+)-DHQ, and (+)-DHM, were synthesized as described before [27], [38] using recombinant F3′5′H from *Sollya heterophylla* and F3′H from *Tagetes erecta*. (±)-Taxifolin hydrate was purchased from Sigma-Aldrich (Munich, Germany).

### Plant material

The studies were performed on pink/blue and white/blue flowering chimeras and white genotypes (arisen after somatic segregation of the white/blue chimera type) of *Angelonia* ×*angustifolia* plants (Figure 1) which were previously described [2]. The plants were grown in the greenhouse at the Julius Kühn-Institut in Quedlinburg (Germany). Flowers were harvested, shock-frozen in liquid nitrogen and stored at −80°C until use.

### Enzyme preparation

Enzyme preparations were obtained using protocols 1 and 2 as described [31]. To remove low molecular compounds, crude enzyme preparations were passed through a gel chromatography column (Sephadex G25, GE Healthcare, Freiburg, Germany). Protein content was determined by a modified Lowry procedure [55] using crystalline bovine serum albumin as a standard.

### Enzyme assays

Assays with enzyme preparations from *Angelonia* petals were performed according to [31] using naringenin as a substrate for FHT and DHQ as a substrate for DFR. CHS cannot be measured independently from CHI because the latter does not need any cofactor and rapidly converts naringenin chalcone formed by CHS into naringenin. Therefore, a combined assay was used as described previously [31]. For the standard enzyme assay with the recombinant DFR obtained from yeast, the reaction contained in a final volume of 50 µL: 15 µL enzyme preparation (22 µg), 0.048 nmol (^14^C)-dihydroflavonol, 0.25 nmol NADPH, 30 µL 0.1 M KH_2_PO_4_/K_2_HPO_4_ buffer pH 6.5 containing 0.4% Na ascorbate. The amount of enzyme was chosen within a range to ensure that the maximum conversion rate of the best substrate was around 50% (linear range of the reaction). The reaction mixture was incubated for 15 min at 25°C. Assays using DHK and DHQ as substrates were stopped by addition of 10 µL 100% acetic acid with products and substrates being extracted twice with 50 µL ethyl acetate. The combined organic phases were transferred to a pre-coated thin-layer cellulose plates without fluorescence indicator (Merck, Darmstadt, Germany) and developed in chloroform/acetic acid/water (10∶9∶1, v:v:v). The evaluation was carried out on a Berthold LB 2842 TLC Linear Analyzer (Wilbad, Germany) by integration of the peak areas. Assays using DHM as substrates were stopped with the addition of 10 µL 100% acetic acid and 50 µL methanol. The mixture was chromatographed on 20 cm×1 cm stripes of paper (Schleicher Schuell, 2043 b, Germany) in chloroform/acetic acid/water (10∶9∶1, v:v:v). Conversion rates were determined using a Typhoon 8600 and the software ImageQuant 5.1 (GE Healthcare, Munich, Germany).

### Characterization of the recombinant DFR

All data represents an average of at least three independent experiments. Determination of the pH optimum was carried out as described for the standard DFR assay, but using 0.2 M McIlvaine buffers with pH values between 4.5 and 9.0.

### Cloning of *DFR* cDNAs from *Angelonia* × *angustifolia*

mRNA was isolated from *Angelonia* petals using the µMACS mRNA Isolation Kit (Miltenyi Biotec, Germany). cDNA was prepared using the SuperScript II Reverse Transcriptase (Invitrogen, Carlsbad, CA) and the oligo(-dT) anchor primer GACCACGCGTATCGATGTCGAC(T)16V. Based on information available in the NCBI-GenBank, nucleotide or amino acid sequences of *DFRs* from other plants from the order Lamiales, were aligned (Accessions X15536; EU305680, Y09127, EF522156) and conserved regions in the N-terminal region adopting the Rossmann fold [28] were used for the design of degenerated primers. The obtained cDNA fragments were isolated, ligated into the vector pCR2.1-TOPO (Invitrogen, Carlsbad, CA) and transformed in *E. coli* strain TOP10 (Invitrogen, Carlsbad, CA). Fragments were sequenced by a commercial supplier (Microsynth Austria AG, Vienna, Austria) and sequences were used for the design of specific 5′- and 3′-primers for the amplification of the ends of the *DFR* by RACE techniques, using the SMART RACE cDNA amplification kit (Clontech, Takara Bio Europe, Saint-Germain-en-Laye, France) according to the manufacturer's instructions. Proofreading amplification of the complete open reading frame was carried out using specific forward and reverse primers (Table S1) and the *Taq/Pwo* Expand High Fidelity PCR System (Roche, Mannheim, Germany).

### Phylogenetic analysis

Multiple alignments were performed with ClustalW [56], [57]. The phylogenetic tree was conducted and bootstrapped with MEGA version 5 [58] using the neighbor-joining method and 1000 replicates.

### Heterologous expression in *Saccharomyces cerevisiae*

Proofreading cDNA amplicons were ligated into the pYES2.1/V5-His-TOPO vector (Invitrogen, Paisley, UK). Sense constructs were isolated and confirmed by sequencing. The vectors harbouring the *DFR* cDNAs *Ang.DFR2 and Ang.DFR1* were transformed into yeast strain INV*Sc*1 using the *Sc*. EasyComp Transformation Kit (Invitrogen, Carlsbad, CA). Preparation of the protein fractions was performed using a modified protocol according to Pompon et al. [59]. Protein fractions were shock frozen in liquid nitrogen and stored at −80°C.

### Heterologous expression in *E.coli*

The plasmid pGEX-6P-1 (GE Healthcare, Munich, Germany) was used for the overexpression of the *A.* × *angustifolia DFRs* as GST fusion proteins. Plasmid pGEX-6P-1 was linearized by double digestion with *Bam*HI and *Eco*RI (Fermentas, Germany). Sticky end *DFR* inserts were generated using sticky end PCR [60] with *Pfu* DNA polymerase (Fermentas, Germany) and the primers listed in Table S1. For each of the *DFRs*, two PCR reactions (PCR 1: A-DFR-FL, A-DFR-RS; PCR2: A-DFR-FS, A-DFR-RL) were performed with diluted pYES2.1 plasmids harbouring the *Ang.DFR2 and Ang.DFR1* as templates. The PCR products were eluted and purified from the agarose gel after electrophoresis. Denaturation and reannealing of an equimolare mixture of the PCR products from the PCR 1 and 2 resulted theoretically in 25% double stranded dihydroflavonol 4-reductase sequences with sticky *Bam*HI (GATC) and *Eco*RI (AATT) ends for direct ligation with T4 DNA ligase (Promega) into linearized pGEX-6P-1. After transformation into *E. coli* strain TOP 10 and isolation of the plasmids, integrity of the vector construct harbouring the dihydroflavonol 4-reductases (pGEX-6P-1::*Ang.DFR2* and pGEX-6P-1::*Ang.DFR1*) was confirmed by sequencing. Single colonies harbouring parent DFRs and mutants were used for heterologous expression in *E. coli* as described [61].

### Purification of GST fusion proteins and removal of GST tag by enzymatic cleavage

GST fusion proteins were purified from the cell extract using Sepharose 4B (GE Healthcare, Munich, Germany) in either a batch procedure or by using GST Spin Traps according to the manufacturer's instructions The DFR was liberated from GST by PreScission protease according to the GST Gene Fusion System Manual (GE Healthcare, Munich, Germany).

### Site-directed mutagenesis

Mutants were generated from pGEX-6P-1 vector constructs harbouring the *DFRs* by use of Q5 Site-Directed Mutagenesis Kit (NewEngland Bioloabs, Vienna Austria). Primer were designed using the NEBase Changer^™^ v 1.01 provided at http://nebasechanger.neb.com. The sequences are given in table S1. The integrity of the constructs was confirmed by commercial sequencing (Microsynth Austria AG, Vienna Austria).

## Supporting Information

Figure S1 **Alignment of the Ang.DFR2 amino acid sequence with DFRs from different plant species.** Red letters indicate maximal consensus.
(DOC)

Table S1 **List of primers used.**
(DOC)

Table S2 **Recovery rates estimated by quantification of radioactivity at the scintillation counter after extraction of 10 µM (^14^C)-labelled dihydroflavonols and leucoanthocyanidins.**
(DOC)
