# Supplementary material for: Isolation of Dihydroflavonol 4-Reductase cDNA Clones from Angelonia x angustifolia and Heterologous Expression as GST Fusion Protein in Escherichia coli
Source: PLoS One. 2014 Sep 19;9(9):e107755. doi: 10.1371/journal.pone.0107755 (PMC4169556; doi:10.1371/journal.pone.0107755)
Supplement: Figure S1 — Alignment of the Ang.DFR2 amino acid sequence with DFRs from different plant species. Red letters indicate maximal consensus. (DOC) [file pone.0107755.s001.doc]

**Figure S1:** Alignment of the Ang.DFR2 amino acid sequence with DFRs from different plant species. Red letters indicate maximal consensus.


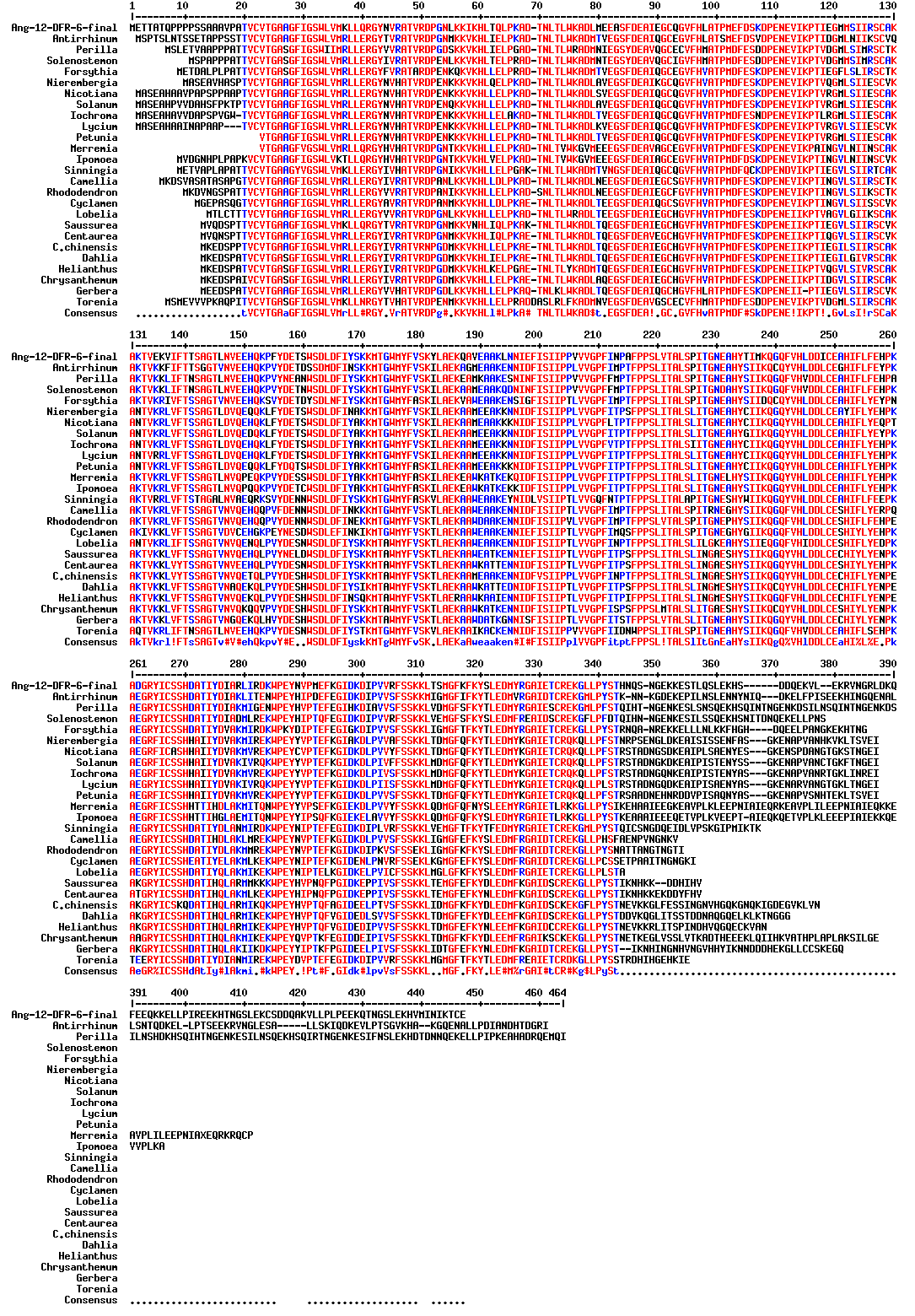


Ang.DFR2

Ang.DFR2

Ang.DFR2

Ang.DFR2
