# Supplementary material for: Isolation of Dihydroflavonol 4-Reductase cDNA Clones from Angelonia x angustifolia and Heterologous Expression as GST Fusion Protein in Escherichia coli
Source: PLoS One. 2014 Sep 19;9(9):e107755. doi: 10.1371/journal.pone.0107755 (PMC4169556; doi:10.1371/journal.pone.0107755)
Supplement: Table S1 — List of primers used. (DOC) [file pone.0107755.s002.doc]

**Table S1:** List of primers used.

| **Primer Name** | **Sequences (5´>3´- direction)** | **T_m_ (°C)** |
| --- | --- | --- |
| Ang.DFR.degF1 | gaycctgagaatgaagtgatcaa | 58.0 |
| Ang.DFR.deg.F2 | tgarggbattgryaaggacatc | 58.0 |
| A-DFRfor2 | CATTTGCTCCTCACATGATGCTACC | 69.4 |
| A-DFRfor3 | TGGCCTGAATATAATGTCCCAATGG | 69.8 |
| A-DFRfor4 | CGACAAGGACATACCGGTGGTG | 70.5 |
| Oligo-dT anchor | GACCACGCGTATCGATGTCGAC(T)_16_V | 76.5 |
| A.DFR.rev 1 | CACCACCGGTATGTCCTTGTCGATG | 73.7 |
| A.DFR.rev 2 | CAGGCCATTTATCTCGGATCAATCTGG | 73.0 |
| A.DFR.rev 3 | GTGAGGAGCAAATGAAGGCGATCC | 72.4 |
| A-DFR-FL | GATCCATGGAGACCACCGCCA | 73.7 |
| A-DFR-FS | CATGGAGACCACCGCCA | 67.4 |
| A-DFR-RL | AATTCCATTTTCTAGATGCTGCCATCA | 69.6 |
| A-DFR-RS | CCATTTTCTAGATGCTGCCATCA | 67.0 |
| RpYES | ACCGGTACGCGTAGAATC | 56.0 |
| A-DFRfullF | ATGGGAGTTACACTTGTGCATG | 63.7 |
| A-DFRfullR | CATTTTCTAGATGCTGCCATCA | 63.9 |
| DFR2_Ang26R_f | ATGTGTCACCaGAGCAGCCGGTTTCATCGG | 73.0 |
| DFR2Ang26R_r | ACGGTGGCGGGGACGGCA | 76.0 |
| DFR2Ang12PP_f | ccaccGTCGGCAGCTGCCGTCCC | 73.0 |
| DFR2Ang12PP_r | TGGTGGTGGTGGTTGTGTGG | 68.0 |
| DFR1Ang26G_f | ATGTGTCACCggcGCAGCCGGTTTCATC | 69.0 |
| DFR1Ang26G_r | ACGGTGGCGGGGACAGCA | 73.0 |
| DFR1Ang12S_f | ACCACCACCAtCGTCGGCAG | 65.0 |
| DFR1Ang12S_r | GGTTGTGTGGCGGTGGTC | 67.0 |
