# Supplementary material for: Isolation of Dihydroflavonol 4-Reductase cDNA Clones from Angelonia x angustifolia and Heterologous Expression as GST Fusion Protein in Escherichia coli
Source: PLoS One. 2014 Sep 19;9(9):e107755. doi: 10.1371/journal.pone.0107755 (PMC4169556; doi:10.1371/journal.pone.0107755)
Supplement: Table S2 — Recovery rates estimated by quantification of radioactivity at the scintillation counter after extraction of 10 µM (14C)-labelled dihydroflavonols and leucoanthocyanidins. (DOC) [file pone.0107755.s003.doc]

**Table S2:** Recovery rates estimated by quantification of radioactivity at the scintillation counter after extraction of 10 µM (^14^C)-labelled dihydroflavonols and leucoanthocyanidins.

|  | recovery rates | | |
| --- | --- | --- | --- |
|  | stripes | organic phase | aqueous phase |
| DHK | 100 | 97 | 3 |
| DHQ | 100 | 97 | 5 |
| DHM | 100 | 95 | 5 |
| Leucopelargonidin | 100 | 92 | 8 |
| Leucocyanidin | 100 | 90 | 10 |
| Leucodelphinidin | 100 | 25 | 20 |

**Figure S1: Alignment of amino acid sequences of DFRs from different plant species. Red letters indicate maximal consensus**.


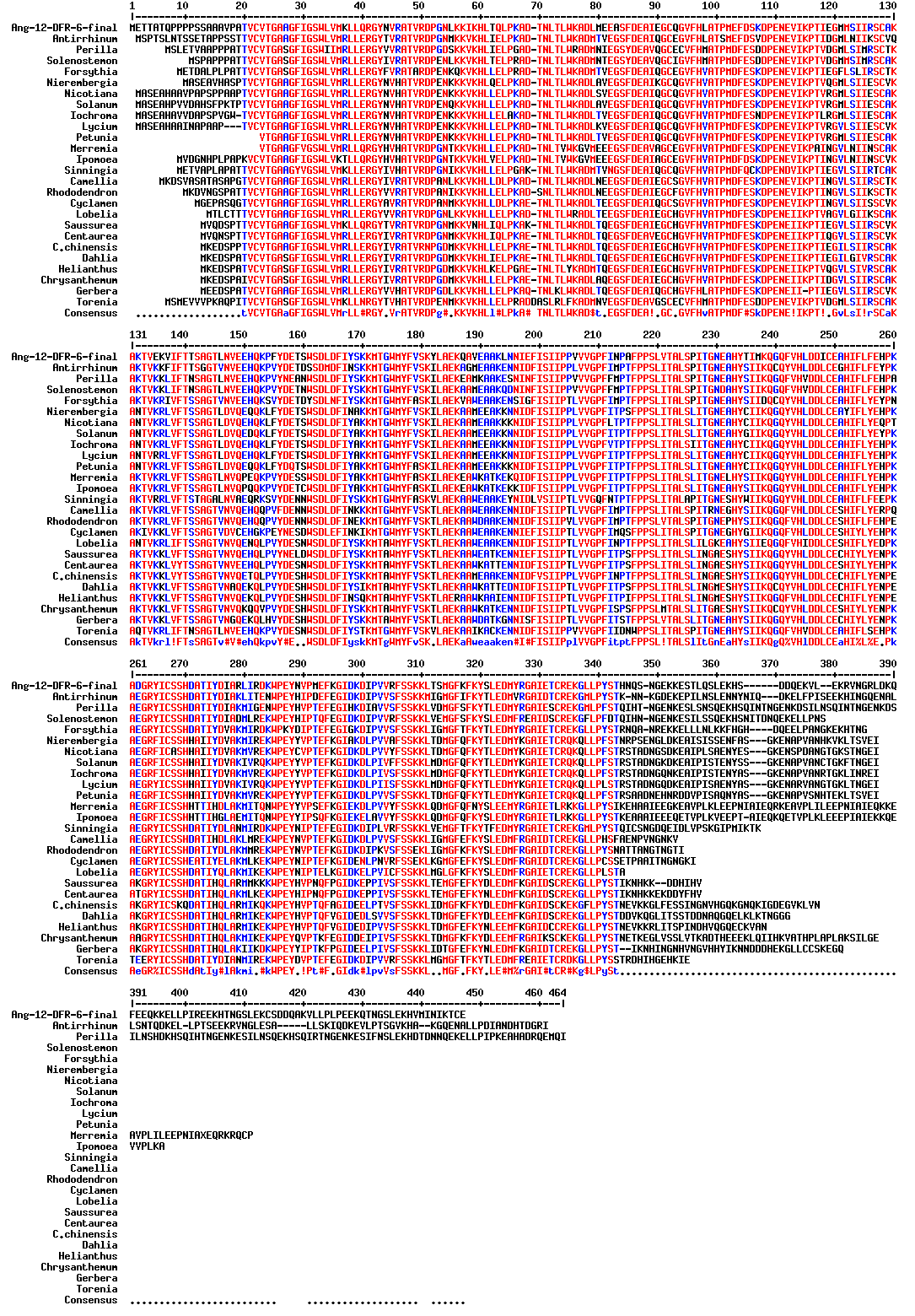


Ang.DFR2

Ang.DFR2

Ang.DFR2

Ang.DFR2
